# Epidemiological approaches in the investigation of environmental causes of cancer: the case of dioxins and water disinfection by-products

**DOI:** 10.1186/1476-069X-10-S1-S3

**Published:** 2011-04-05

**Authors:** Manolis Kogevinas

**Affiliations:** 1 Centre for Research in Environmental Epidemiology (CREAL), Barcelona, Spain; 2 IMIM (Hospital de Mar Research Institute), Barcelona, Spain; 3 CIBER Epidemiologia y Salud Pública (CIBERESP), Spain; 4 National School of Public Health, Athens, Greece

## Abstract

I will refer in this paper to difficulties in research in environmental causes of cancer using as examples research on dioxins and on drinking water disinfection by-products (DBPs) that have created considerable controversy in the scientific and wider community. Dioxins are highly toxic chemicals that are animal carcinogens. For many years, evaluation of the carcinogenicity of dioxins in humans was based on case-control or registry based studies. The development of methods to measure dioxins in blood indicated that these studies suffered from extreme exposure misclassification. The conduct of large cohort studies of workers with widely contrasted exposures together with the use of biomarkers and models for exposure assessment, led to convincing evidence on the carcinogenicity of dioxins in humans. The high toxicity of a few dioxin congeners, the availability of a scheme to characterize the toxicity of a mixture of dioxins and related compounds and the long half-life of these compounds facilitated epidemiological research. Contrary to dioxins, trihalomethanes (THMs) and most of the hundreds of DBPs in drinking water are chemicals of low toxicity. For more than 15 years, the main evidence on the carcinogenicity of DBPs was through ecological or death certificate studies. More recent studies based on individual assessment confirmed increases in bladder cancer risk. However even those studies ignored the toxicological evidence on the importance of routes of exposure to DBPs other than ingestion and, probably, underestimated the risk. Persistence of weak study designs together with delays in advanced exposure assessment models led to delays in confirming early evidence on the carcinogenicity of DBPs. The evaluation of only a few chemicals when exposure is to a complex mixture remains a major problem in exposure assessment for DBPs. The success of epidemiological studies in identifying increased risks lies primarily on the wide contrast of exposure to DBPs in the general population that overcomes the significant exposure misclassification. Exposure assessment has been the Achilles heel for studies on dioxins and DBPs and cancer. The combination of powerful study designs, advanced exposure assessment together with a better understanding of mechanisms of disease and the use of biomarkers of exposure, led to the strengthening of the epidemiological evidence.

## Background

The significant and rapid changes in cancer incidence in the last decades can only be attributed to equally large changes in population exposure to environmental factors. Several environmental exposures are known to cause cancer. Frequently, however, hypotheses linger on for years or decades without being able to provide convincing evidence in one or the other side. This has led to serious criticisms towards environmental epidemiology [1].

The environment defined in a wide sense refers to all factors that are not genetic, and includes lifestyle factors such as tobacco smoking, biological agents such a HPV, medicaments, nutrition, occupational exposures and other. Environment in a more restricted definition includes all non-genetic factors that are not directly controlled by a person or that do not depend on direct choices of the persons. Using the restricted definition active smoking should not be classified as environmental, while second-hand-smoke should. This rather ambiguous definition complicates the attribution of causation to specific risk factors. For example, use of hair dyes is generally considered as lifestyle while exposure to phthalates through the use of hairsprays, is considered environmental. In addition, what frequently are defined as personal choices, for example whether you smoke, are dependent on a variety of factors related to the social and environmental milieu of each person. With these limitations in mind, I will refer in this paper to environmental causes of cancer using the restricted definition of environment, identify major problems in environmental cancer research and discuss how these problems have been overcome. I will use as examples cancer research on dioxins and on drinking water disinfection by-products that have created considerable controversy in the scientific and wider community.

## Environmental causes of cancer

Doll and Peto [2] in their influential and clairvoyant review on the causes of cancer identified a small list of environmental exposures. Their review incorporated environmental exposures that were investigated at that time and that were relatively few. Among the main factors reviewed were air-pollution (PAHs, and arsenic, asbestos, radioactive elements), drinking water (chlorination and fluoridation), food pollution (pesticides), industrial products (hair dyes and other) and geophysical factors (ionizing and solar radiation). Their estimate on the risk attributable to these exposures (the proportion of disease caused by these factors) is unclear and is possibly less than 3% to 4%. Research since the early 1980s has provided extensive new evidence on environmental exposures associated with cancer (Table 1). Since the 1980s, we have accumulated conclusive proofs on the carcinogenicity of some of the exposures shown in Table 1 such as arsenic in water [3]. Some major exposures currently investigated did not occur at the time of the review by Doll & Peto, as is for example the population exposure to radiofrequencies through the massive use of mobile phones. There are no generally accepted comprehensive overall estimates of the attributable risk for environmental cancer. Estimates for specific risk factors differ considerably [4,5].

**Table 1:** Environmental exposures associated with cancer. The strength of the association differs and convincing evidence is available only for some of the exposures

• Air pollutants
– Outdoor air e.g. particulates, PAHs, asbestos, erionite, benzene
– Indoor air e.g. second-hand-smoke, combustion wood, combustion coal, high temperature frying, formaldehyde
• Water contaminants e.g. arsenic, disinfection byproducts, nitrates
• Persistent Organic Pollutants, e.g. dioxins, PCBs, endocrine disrupters, food contaminants
• Metals, e.g. arsenic, chromiumVI, cadmium
• Industrial products e.g. hair dyes
• Radiations
– Ionising (radon)
– Solar radiation
– Electromagnetic Fields (EMF), extremely low frequency and radiofrequencies

**Figure 1:**
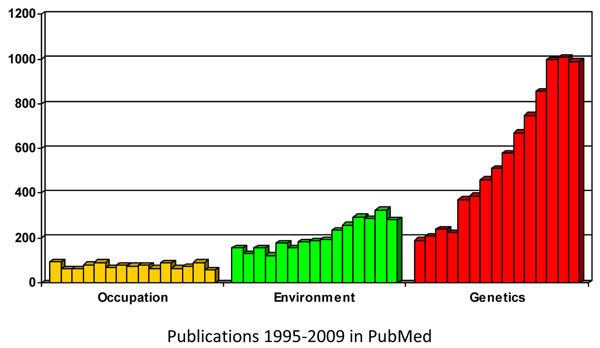
Time trends in publications in occupational, environmental and genetic cancer epidemiology 1995-2009 (no limits, hits retrieved from PubMed)

The volume of research in environmental causes of cancer has been growing. Time trends in research in occupational, environmental and genetic cancer epidemiology are shown in Figure 1 based on data retrieved from PubMed for the period 1995-2009 (no language limitations). Publications in occupational cancer epidemiology have remained stable to around 75 per year, though this means that there has been a proportional decline given the overall increasing time trends in scientific publications. Publications in environment cancer epidemiology have doubled from around 150 publications per year in the mid 1990s to approximately 290 fifteen years later. This increase is similar to the average increase in all scientific publications listed in PubMed that doubled during these 15 years from around 430,000 in 1995 to 815,000 in 2008. The increase of papers in environmental cancer epidemiology is small compared to an unprecedented increase in genetic cancer epidemiology that quadrupled in these fifteen years with an average of around 1000 papers per year at end of the period.

Whether the lack of a major increase in publications in environmental cancer as compared to genetic cancer epidemiology should be evaluated as an expression of crisis in this field at a time when there is continuous and growing interest on the environment in general, is outside the scope of this paper. Whatever the overall evaluation may be, it is clear that certain of the environmental causes of cancer examined have created considerable controversy, e.g. extremely low frequency electromagnetic fields (EMF ELF), mobile phones, dioxins, and other and have frequently not led to solid conclusions and public health action, or led to action after long delays.

## Dioxins and cancer: the epidemiological evidence

Dioxins and related compounds are unwanted chemical agents that are produced in several industrial processes such as the incineration of municipal waste, processes in the pulp and paper industry or the metal recycling industry. In the 1970s, dioxins were contaminants of the widely used phenoxy herbicides such as Agent Orange. There exist 210 polychlorinated dioxin and furan congeners. 2,3,7,8 tetrachlorodibenzo-p-dioxin (TCDD) is the most toxic compound of this family of structurally related chemicals that have a common mechanism of action and are believed to induce the same spectrum of health related effects. TCDD is considered by the IARC as a human carcinogen since 1997 [6] and it was recently re-evaluated and again classified as Group 1 carcinogen [7] based on sufficient evidence on both humans and animals. The WHO/FAO (World Health Organization/ Food and Agriculture Organization) recommended in 2002 [8] that human ingestion in adults should stay below a limit of 70 pg/kg weight/month that corresponds to approximately 2pg/kg per day. This limit is relatively close to the average intake of several populations although in recent years considerable decreases have been achieved in several industrial countries such as the USA and Germany. Critical effects used to define this Tolerable Daily Intake (TDI) were effects on the reproductive, developmental and endocrine systems. Cancer effects in animals are multiple and well characterized but appear at higher doses. Modelling of epidemiological data have identified increased cancer risks at doses only few times higher than the TDI set by WHO/FAO [9]. Exposure to humans is nearly entirely through the diet, particularly milk and other dairy products, fish and meat. The case of dioxins is of interest for research because it refers to a highly toxic chemical that produces multiple cancers in animals for which, however, epidemiological studies had serious difficulties in identifying cancer risk in humans. This led for a long period to a controversy that was finally solved in the wider scientific community (with few exceptions of sceptics) when appropriate study designs and appropriate exposure methods based on combination of modelling and biomarkers provided solid evidence [10].

The similarity of action of different dioxins, has led to the development of a relative potency-ranking scheme using toxic equivalent factors (TEQ). The total dioxin-like activity of a complex mixture is expressed as the weighted sum of all the dioxin-like chemicals [11]. This scheme includes 17 dioxins and furans and a small number of PCBs that show dioxin-like activity. Dioxins are lipophilic, are slowly metabolized and eliminated, and tend to bioaccumulate. The half-life of different compounds differs, but the TEQs of the mixtures to which humans are exposed are usually driven by just a few of these compounds. The half-life of TCDD has been estimated in humans to be between 7 and 8 years. Most of the effects of dioxins are believed to be mediated through the Ah receptor that is highly conserved in different species. Various dioxin effects including enzyme induction, immunotoxicity, developmental effects, tend to be similar irrespective of whether the exposure is acute or chronic and this probably reflects the fact that it is the tissue concentration which is directly associated with the response.

The epidemiological studies on dioxins include those of industrial exposures in workers producing phenoxy herbicide and chlorophenols, studies of the population exposed in the industrial accident in Seveso, studies of subjects exposed during herbicide application particularly, application cohorts of military personnel of the US army in Vietnam, commercial application cohorts, and community based studies (case-control studies).

For many years the discussion on the carcinogenicity of dioxins in humans were based on results from community-based studies or on studies from registries including pesticide applicators. Findings on cancer risk among subjects evaluated in community based studies and sprayer applicator studies are contradictory. The large discrepancies observed are mostly due to exposure misclassification, since most subjects classified as exposed in those studies had probably very similar or only slightly elevated levels of TCDD compared to those classified as non-exposed.

**Figure 2:**
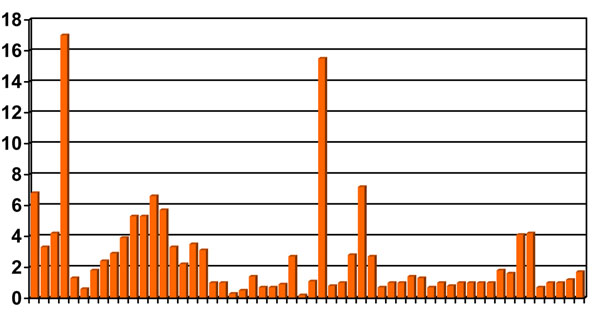
Odds Ratios for soft-tissue sarcoma and exposure to phenoxy herbicides, chlorophenols and dioxins. 15 case-control studies (adapted from E Johnson 1993, ref. [34])

Figure 2, shows the ORs for around 15 case-control studies evaluating exposure to phenoxy herbicides, chlorophenols and dioxins and soft-tissue sarcoma risk. A similar pattern is observed for lymphomas. The first study on phenoxy herbicides and soft-tissue sarcoma that is a rare tumour in adults was done in northern Sweden [12]. Although the methods of that study were criticized, findings were later replicated in other studies in Sweden [13]. However, when evaluating all available studies, the overall picture of risk was far from being conclusive with many discrepant findings (Figure 2). This created significant scepticism in the scientific community.

The difficulties in evaluating exposure in users of pesticides and long-term effects are well known (14). Until recently, the limited knowledge on effects of pesticides on cancer comes mostly from industrial cohorts of workers producing a specific compound rather than from studies of users. The main problem in studies on users of pesticides, professional or the general public is the extreme difficulty in identifying exposure retrospectively given the variety of compounds used in agriculture and the lack of knowledge of specific compounds by the users. Although several elaborate methods have been developed to identify exposure to pesticides in case-control studies [14], this remains a very difficult task. If exposure to a parent compound (pesticide) is difficult to evaluate, it is practically impossible to evaluate exposure to a contaminant of a pesticide, such as dioxins that are contaminants of specific phenoxy herbicides and chlorophenols.

**Figure 3:**
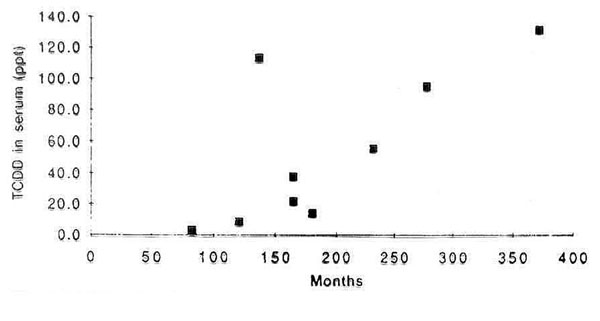
Concentration of TCDD in serum of New Zealand professional sprayers (applicators) in relation to total months spent spraying 2, 4, 5-T (Smith et al, JNCI. 1992, ref. [15])

The development of an assay to measure dioxins in blood lipids, allowed an evaluation of exposure to these compounds in the worker and general populations. Analyses among professional sprayers (applicators) of phenoxy herbicides [15] showed that their exposure was far lower than that of production workers of the same compounds. In sprayers of phenoxy herbicides in New Zealand, it was shown that only prolonged exposures of more than 10 years would unequivocally increase TCDD levels of the sprayers to levels above background (Figure 3). This meant that the assessments done in case-control studies grossly misclassified exposure to dioxins. In many studies, the cut-off to identify someone as exposed to dioxins would have been the use of phenoxy herbicides for periods as short as 1 week or 1 month. Studies using measurements of TCDD in blood indicated that this exposure would not be associated with any measurable increase in exposure to dioxins.

The development of assays to measure TCDD and other dioxins and the decision to study prospectively industrial cohorts and accidentally exposed populations (Seveso) gave conclusive findings concerning the carcinogenicity of dioxins. There has been considerable effort in characterizing the exposure of these populations, which are exposed from 10 to 1000 times higher levels of TCDD than the general population. Cancer mortality was invariably increased in all industrial cohorts examined (Table 2). Statistically significant moderate increases of the order of 40% to 50% were observed among the exposed subcohorts of these populations and higher risks were observed for subjects with the highest exposures. Positive linear trends in risk were found with increasing exposure for all cancers combined and for some specific cancers [16]. Increased risks with time since first exposure were observed in those studies that evaluated latency [17].

**Table 2:** Mortality from all neoplasms in selected industrial cohorts with high exposure levels to PolyChlorinated Dibenzo Dioxins and Furans (adapted from IARC 1997, ref [6]).

Reference	No. deaths	SMR (95% CI)
**IARC International cohort**		
Kogevinas et al 1997, ref [29]	394	1.2 (1.1-1.3)
**Industrial populations (high exposure sub-cohorts)**		
Steenland et al 1999, ref [30]	40	1.6 (1.2-1.8)
Becher et al 1996, ref [31]	105	1.3 (1.0-1.5)
Hooiveld et al 1997, ref [32]	51	1.5 (1.1-1.9)
Ott & Zober 1996, ref [33]	18	1.9 (1.1-3.0)

In examining the findings on cancer risk from the most informative epidemiological studies (informative in terms of validity of exposure assessment and inclusion of subjects with high dioxin exposure) a number of issues should be noted. Low excess risks for all neoplasms combined were found in all industrial cohort studies with adequate exposure assessment that applied biomarkers and modelling. These excess risks were highly statistically significant and an effect of chance can be excluded. The risk tended to be higher for those workers with the highest exposures. Risk for several specific cancers were increased in some of these studies (lymphomas, multiple myeloma, soft-tissue sarcoma, lung cancer, liver cancer, breast cancer, testicular cancer, endometrial) but consistent findings were only found (apart from all neoplasms combined), for lung cancer, lymphomas and soft-tissue sarcoma [7]. At present, the real dilemmas are not whether dioxins are or not carcinogens, but rather on the quantification of the risk associated with the very low-level exposure of the general population.

## Dioxins and cancer in humans, why did it take so long to conclude?

The first case-control studies were conducted in the late 1970s [12]. It was only in 1997 that IARC classified TCDD as human carcinogen based on limited evidence in humans and very strong evidence in animals together with mechanistic information. At that time, even though IARC classified dioxins as carcinogens there was widespread discussion about whether this classification was appropriate. It was only until 2009 that IARC in a new evaluation classified TCDD as carcinogen also based on sufficient human evidence. In addition, in 2009 a number of specific sites were identified to be specifically related to TCDD apart from an overall cancer increase.

The long delay in providing conclusive evidence was due to wrong study design and to extreme exposure misclassification. Notwithstanding the importance of the first case-control studies in identifying the problem, this design appeared inadequate to identify increased risks in the general population for a contaminant of pesticides such as dioxins. The reason was that differences in exposure to TCDD in the general population can be expected to be small with only very few subjects having high exposures and the tools used in the case-control studies are inadequate to identify these differences. This does not mean that exposure even at low levels cannot produce cancer; it simply means that the research design used could not correctly identify these risks. Extreme exposure misclassification occurred due to very low exposure levels and the lack of contrast in exposure of the general population. Use of contaminated phenoxy herbicides in the backyard may result to exposure to dioxins, but this is minimal and impossible to capture with questionnaires.

We were finally successful in identifying the cancer risks associated with TCDD because of (i) application of appropriate designs and exposure-assessment methods; and (ii) some inherent characteristics of dioxins that facilitated the evaluation of the risks.

The research aspects concerning the success are mainly the conduct cohort studies in populations that included subjects with widely contrasted levels of exposure to TCDD and the development of biomarker that allowed measurement of levels of TCDD and other dioxin –like compounds in serum samples of large populations.

Some of the characteristics of the toxicity of these compounds also made possible this research. The first is that some of the dioxins and furans are highly toxic chemicals and TCDD is among the most toxic compounds tested in the laboratory. In addition, for dioxins we have a usable scheme for the measurement of the toxicity of a mixture that has been developed based on extensive information on the toxicity of many of the individual dioxins, furans and dioxin-like PCBs. Given that our exposure is to mixtures rather than to single chemicals, exposure assessment should be done on the mixture rather than on one single chemical. The TEQ system allowed this evaluation of total toxicity. In this case, the identification of a small set of very toxic chemicals can characterize well the toxicity of a mixture. The second characteristic that helped research (and that relates to the high overall toxic effects of these chemicals) is their long half-life that for TCDD is around 7 years. Chemicals with long half-life can be measured many years after exposure has stopped and still allow an evaluation of level of exposure in the past through the use of models.

## Drinking water disinfection by-products and cancer in humans

Drinking water disinfectants include highly reactive molecules that generate undesired compounds through reaction with organic matter. These disinfection by-products (DBP) constitute complex mixtures of chemical species with different properties. Chlorine, the most widely used disinfectant for drinking water, gives rise to trihalomethanes (THM), that in most waters are the most prevalent DBPs. Some epidemiological studies have shown an association between long-term exposure to chlorination by-products and increased risk of cancer which is supported by experimental evidence of carcinogenicity for some of these chemicals [18]. Many of these compounds have been shown to be genotoxic, but the mechanisms of action are not well elucidated and few studies have evaluated markers of genotoxicity in humans.

**Figure 4:**
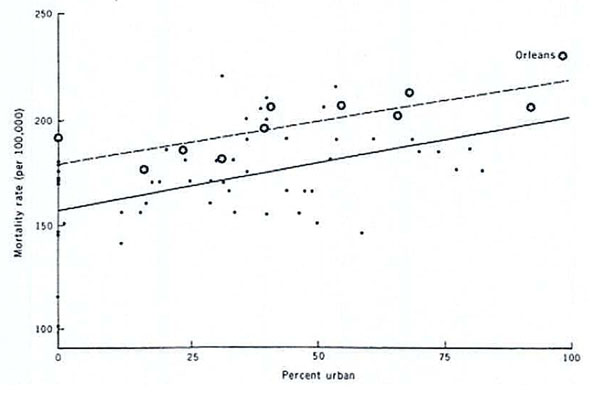
Total cancer mortality and urbanization for parishes without drinking water from the Mississippi (closed circles) and those with some al all water from the Mississippi (stars). The latter are expected to have higher exposure to THMs (from Page et al 1976, ref. [19])

**Figure 5:**
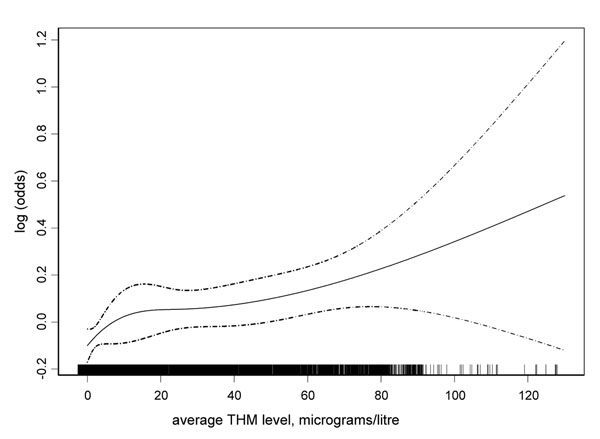
Pooled analysis of case-control studies on bladder cancer and exposure to Trihalomethanes in drinking water. Log odds ratio for bladder cancer and average exposure to THMs (mg/L) using natural splines (3df), both sexes (from Villanueva et al 2004, ref. [22])

THMs in drinking water were first identified in 1974. Concerns were expressed shortly after, regarding potential health effects of these compounds given their toxicity in experimental animals. The first studies were ecological comparing levels of THMs (or proxies) with cancer mortality and suggested bladder as one of the cancer sites associated with chlorinated water intake. For example, a comparison of mortality in different areas around the Mississippi river [19] indicated that cancer mortality was higher in more urban areas and that it was higher in areas getting water from the river where higher levels of THMs could have been expected (Figure 4). Several similar studies were published in the following years and when the International Agency for Research on Cancer evaluated chlorinated drinking water as a potential human carcinogen in 1991 [20], most of the available studies were ecological or death certificate-based. These studies typically used cross-sectional estimates of exposure usually around the time of death, and were limited in their ability to adjust for other risk factors. The methodological limitations led IARC to conclude that the evidence for the carcinogenicity of chlorinated drinking water in humans was inadequate (group 3). After this evaluation, several studies with improved exposure assessment at the individual level were published. Among them, the studies of bladder cancer reported positive associations with chlorination by-products exposure. A meta-analysis of studies on bladder cancer [21] with individual information on residence and water consumption reported an increased risk in subjects with long-term consumption of chlorinated drinking water. A posterior pooled analysis [22] confirmed these findings and reported an increased risk for levels that occurred normally in industrial societies (Figure 5). These analyses were published about 30 years after the identification of THMs and provided the strongest evidence for an effect of DBPs on cancer.

While ingestion considered as the main route of exposure to DBPs, the high volatility and dermal permeability of certain DBPs leads to a significant contribution of the inhalation and dermal absorption pathways. Experimental studies have shown a significant uptake of THMs through these routes when showering, bathing or swimming in pools [23,24]. The Spanish bladder cancer study [25] was the first study to evaluate multiple routes of exposure by requesting detailed information on activities such as baths, showers, swimming pool attendance that would lead to exposure by non-ingestion routes. In this study, THM levels were used as marker of DBP exposure. An increased risk was associated with both ingestion and inhalation/dermal absorption, but the latter routes led to higher contrasts in risk (Table 3). What was further shown in this study was that subjects living in areas with poor quality water and very high levels of THMs tended to consume preferentially bottled water that does not have THMs [26]. Among the subjects who would have been classified as no- or very low-exposed to THMs had the evaluation been focused only on ingestion, 45% would have been classified as high or very high exposed through showers and baths. The non-exposed group, therefore, included subjects not exposed by ingestion, but who were heavily exposed in showers and baths through inhalation and dermal absorption. There is still no consensus on the cancer risk of exposure to DBPs/THM, but there is accumulating evidence particularly for bladder cancer.

**Table 3:** Odds ratios (OR) and 95% confidence intervals (CI) of bladder cancer for different indices of exposure to disinfection by products from a hospital-based case-control study conducted in Spain (adapted from Villanueva et al 2007, ref. [25])

Quartiles of exposure	Ingestion	Inhalation/Skin absorption
	OR (95%CI)	OR (95%CI)
First (Lowest)	1.0 (ref)	1.0 (ref)
Second	0.9 (0.6-1.3)	1.3 (0.9-1.9)
Third	1.2 (0.8-1.7)	1.4 (0.9-2.1)
Fourth (highest)	1.4 (0.9-2.0)	1.8 (1.2-2.9)
p-value for linear trend	0.09	<0.01

## Disinfection by-products and cancer in humans, why does it take so long to conclude?

Similar to dioxins, the difficulty in providing conclusive evidence for an association between exposure to DBPs and cancer in humans lies both in inherent characteristics related to the toxicity of these compounds and in issues regarding the epidemiological methods used.

Contrary to dioxins, DBPs are chemicals of low toxicity, at least as evaluated in experimental studies. There are about 700 DBPs identified in drinking water and most are in minute concentrations. Epidemiological studies have focused on THMs, because they are carcinogenic in experimental animals and because these they were the most prevalent DBPs in most waters. However, short term toxicity studies indicate that THMs are not among the most toxic DBPs compared to other chlorinated DBPs (e.g. MX), or to brominated and iodinated DBPs [27]. A complication in the evaluation of the toxicity of mixtures of DBPs in waters is that none of the prevalent DBP is much more toxic. The result is that drinking waters that have a mixture of hundreds of DBPs cannot be easily characterized for their toxicity through the identification of one or very few specific DBPs. As much as knowledge accumulates on the toxicity of numerous DBPs, it appears that the toxicity of a water sample measured through the evaluation of THM levels would provide only a small fraction of the total toxicity. Mixtures have, by definition, variable composition in time and space and significant differences in water contaminants between areas have repeatedly been shown. Until now, there exists no toxicity algorithm similar to that of PAHs or dioxins to calculate toxicity of mixtures. Finally these chemicals have short half-lives, so unless measurements in biological samples are done immediately (e.g. in exhaled breath or in other media) they cannot be identified *per se* or by measuring metabolites. This scenario of relatively low toxicity chemicals with short half-lives that occur in mixtures is the norm rather than the exception in studies in environmental cancer.

The problems with the epidemiological research on DBPs related to the type of study designs used for many years and the poor exposure assessment that was further complicated due to the ignorance of toxicological evidence on routes of exposure to DBPs. For more than 15 years, the main evidence on the carcinogenicity of DBPs was through ecological or death certificate studies. We now know that because of the importance of non-ingestion routes of exposure, the water that enters the house may largely determine the exposure of inhabitants of a house. Therefore, an ecological based exposure evaluation may provide a less misclassified assessment compared to circumstances with higher variability of exposure within small areas. However, these studies still carry an uncertain degree of exposure misclassification because of the lack of detailed information of exposure at the area and individual level, and the cross sectional nature of the studies that usually refer to the last residence only. They have also the other problems of ecological studies such as unmeasured confounding. Death certificate studies share the same problems. The fact that ecological studies indicate clearly an increase in bladder cancer (and not of many other cancers) point to a real effect. However, reliance only on ecological and death certificate studies to evaluate risks that are not very high, cannot but create distrust about the findings. When the evidence is repeatedly based on relatively weak study designs and no stronger evidence is provided, then unavoidably this may create distrust. The first well-conducted individually based study was published in the 1987 [28], and the first study to take into account exposure in a more comprehensive way, published in 2007 [26]. Given the widespread exposure and the high prevalence of the cancer involved, this appears as a gross error in research priorities: if the risk is real then we should prevent it, and if it is not real, then we should verify this and avoid taking measures.

Toxicological studies indicated since the late 1980s that inhalation and dermal absorption for many DBPs were important exposure routes. This evidence took around 10 years to reach epidemiologists and be implemented in our protocols. This delay is probably due to the existence of a certain compartimentation in science that complicates the transmission of knowledge across scientific areas. Inclusion of questions on different tasks that provide information on different exposure routes, make the epidemiological assessment much more complete. As mentioned above, not evaluating other exposure routes may lead to the definition of persons drinking bottled water as non-exposed while they are in reality exposed by inhalation or dermal absorption [26]. This may lead to considerable misclassification since areas with poor quality water are frequently those where there is high consumption of bottled water. The whole picture may be more complicated since not all compounds are volatile and not all are absorbed through the skin. The second most common DBPS (haloacetic acids) are non-volatile and the route of principal exposure is ingestion. In addition, these chemical are found in high concentrations in swimming pools since they do not volatilize.

The evaluation of only a few chemicals when exposure is to a complex mixture is problematic. In some waters, THMs could be used as marker chemicals for the whole mixture but this should be verified through chemical analyses of different compounds in the water. The hypothesis that the toxicity of the whole mixture can be accurately estimated by measuring THMs remains an assumption and may lead to significant misclassification. Alternatives to this assumption are not obvious particularly for long-term retrospective studies since extensive routine measurements in water are only available for THMs, and even these are scarce.

The main reason why significant misclassification in exposure has not impeded several epidemiological studies to identify risks is that there are wide contrasts of exposure in DBP levels between populations. Even significant misclassification still allows an adequate identifies populations with widely different levels of exposure to THMs (and presumably other DBPs). In prospective studies, the measurement of a wide range of chemicals is feasible as also may be the use of global unspecific tests for toxicity, such as the Comet assay or mutagenicity in waters using the Ames test.

Future studies should be large, combine ecological and individual information on exposure, evaluate multiple chemicals and not only THMs and evaluate multiple routes. In a situation of relatively low risks, even relatively small improvements in exposure assessment may make significant differences in the studies. The use of new evidence on mechanisms of these compounds including use of genotyping to evaluate effect modification should be promoted.

## Conclusions

In recent years, large studies have been conducted focusing on a variety of environmental exposures. The identification that exposure assessment has been the Achilles heel for studies in environmental cancer has led to the use of more advanced techniques for exposure assessment and to considerable improvements in the evaluation of exposure. These involve the use of classical environmental epidemiological techniques and in recent years the use of modern techniques such as satellite imaging etc. In addition, these advances have gone along with more powerful statistical analyses. Cohort studies have applied biomedical technology and have used more extensively biomarkers of exposure. The better understanding of mechanisms of disease has finally allowed the development of more accurate hypotheses. The issue of evaluating mixtures and particularly mixtures of low toxicity chemicals remains difficult to solve and certainly requires the application of state-of-the art methods to minimize inherent difficulties in the detection of risks associated with exposure to such chemicals. It also will require extensive discussion to evaluate whether modern biotechnology can provide new methods for the characterization of this toxicity. I used two examples of the evolution of evidence on the carcinogenicity of dioxins and of water DBPs. I showed that the combination of more powerful study designs and more accurate exposure assessment together with a better understanding of mechanisms of disease and the use, in the case of dioxins, of biomarkers of exposure, led to the strengthening of the epidemiological evidence.

## Competing interests

The author declares that he has no competing interests.
